# Using SWE Standards for Ubiquitous Environmental Sensing: A Performance Analysis

**DOI:** 10.3390/s120912026

**Published:** 2012-08-31

**Authors:** Alain Tamayo, Carlos Granell, Joaquín Huerta

**Affiliations:** 1 Institute of New Imaging Technologies, Universitat Jaume I, Av. Vicent Sos Baynat, SN, 12071, Castellón de la Plana, Spain; E-Mail: huerta@uji.es; 2 European Commission, Joint Research Centre, Institute for Environment and Sustainability, Via E. Fermi 2749, I-21027 Ispra (VA), Italy; E-Mail: carlos.granell@jrc.ec.europa.eu

**Keywords:** sensor web, environmental modelling, smartphone applications, performance modelling, sensor web enablement, XML, EXI, JSON

## Abstract

Although smartphone applications represent the most typical data consumer tool from the citizen perspective in environmental applications, they can also be used for in-situ data collection and production in varied scenarios, such as geological sciences and biodiversity. The use of standard protocols, such as SWE, to exchange information between smartphones and sensor infrastructures brings benefits such as interoperability and scalability, but their reliance on XML is a potential problem when large volumes of data are transferred, due to limited bandwidth and processing capabilities on mobile phones. In this article we present a performance analysis about the use of SWE standards in smartphone applications to consume and produce environmental sensor data, analysing to what extent the performance problems related to XML can be alleviated by using alternative uncompressed and compressed formats.

## 1. Introduction

Changes in environmental conditions affect the environment itself and put pressure on human society [1]. Tools for modelling, monitoring, and assessment have increasingly become crucial instruments to continually observe the environment and the Earth. In this context, the GEOSS initiative (*Global Earth Observation System of Systems*) pursues to connect data producers of environmental and sensor data, and decision-support tools with end users, with the aim of exploiting the potential of Earth observations and sensor data to tackle with global issues [2]. GEOSS is regarded as a global “system of systems” that includes sensor networks, data communication protocols, spatial-environmental data infrastructures and other essential components and technologies for monitoring and observing the Earth. The combination of sensor networks (data providers), smartphone applications (data consumers), along with data communication protocols, forms the basic pieces in most ubiquitous environmental sensing applications.

Nowadays, smartphone applications represent a typical data consumer tool from the citizen perspective, largely motivated by the rapid increase of hardware capabilities of these devices, which have permitted an exponential growth in the number of applications targeted to them. As a result, smartphones not only play a traditional role as consumers of sensor-related information but also may act as producers of such information. As they are increasingly equipped with a variety of data-capturing sensors (ambient light sensors, accelerometer, digital compass, gyroscope, GPS, proximity sensor, microphone and cameras), smartphone applications are becoming attractive in many scenarios [3] such as health [4,5], traffic [6,7], or environmental monitoring [8,9]. In addition to sensors included on the phones, external devices can be attached to them to track dynamic information about different phenomena [10]. For the consumer role, environmental sensor data can be retrieved from remote sensors through such applications. For the producer role, people can make environmental observations with these devices and share them with other users. For example, smartphones are being used for *in-situ* data acquisition in varied scenarios such as geological sciences [11,12], epidemiology [13], biodiversity [14], and noise pollution monitoring [8,9]. In these examples smartphones play either a consumer or producer role as typical clients in a client-server architecture. Nevertheless, they may also act as intermediaries or client aggregators. For instance, in low-connectivity situations, a mobile application may consume and process data from nearby *in-situ* sensors and upload aggregated datasets to the corresponding servers when network links are restored [15–17]. In this particular case, smartphones may potentially collect large quantities of data to be further uploaded to remote servers, which may be a serious impediment in terms of performance.

Providers and consumers exchange sensor data through communication protocols. Internet and Wireless Sensor Networks (WSN) are examples of active communication channels that connect sensor networks and consumer applications. Regardless of the particular channel chosen, communication is based on internationally adopted standard protocols [18]. The use of standard protocols to exchange information between smartphones and sensor infrastructures (servers, services, *etc.*) brings several benefits to both developers and users such as interoperability and scalability. In this context, the Open Geospatial Consortium (OGC) (http://www.opengeospatial.org) has developed a set of standards to deal with sensor-related data. These standards are part of the Sensor Web Enablement (SWE) initiative that aims to provide data communication protocols via XML-based encodings and service interfaces for discovering, accessing and exchanging any kind of sensor data [19]. The use of XML increases network traffic as a consequence of its well-know verbosity [20–25], which may seriously affect performance on the client side, issue that is particularly important for smartphones because of their limitations related to bandwidth, loss of connectivity, smaller screen size, and battery life.

Motivated by the need of using standard protocols and, at the same time, avoiding or reducing communication overhead, we present in this article a performance analysis about the use of SWE standards as data communication protocols in smartphone applications to consume and produce environmental sensor data. We analyse to what extent the performance problems related to transmitting and processing potentially large messages encoded using XML can be alleviated by using alternative uncompressed and compressed formats. Our experiments are based on an ample set of sensor datasets in terms of size and role (*data consumption vs. provision*) and the usage of different encoding formats. In addition, we perform our experiments with two different types of mobile terminals and over two types of communication links (Wi-Fi, 3G).

The remainder of this article is structured as follows. Section 2 introduces topics such as Sensor Web Enablement and some of its specifications, as well as the main exchange formats considered in this study: XML, JSON and EXI. Section 3 provides a wide review of related work. After this, Section 4 introduces selected datasets, materials used, and the methodology followed to conduct our experiments. Results are thoroughly discussed in Section 5. Last, Section 6 presents conclusions of our work.

## 2. Background

The *Sensor Web Enablement* (SWE) initiative is a framework that specifies interfaces and metadata encodings to enable real-time integration of heterogeneous sensor networks. It provides services and encodings to enable the creation of web-accessible sensor assets [26]. SWE is an attempt to define the foundations for the *Sensor Web* vision, a worldwide system where sensor networks of any kind can be connected [27–29]. It includes specifications for service interfaces such as *Sensor Observation Service* (SOS) [30] and *Sensor Planning Service* (SPS) [31], as well as encodings such as *Observations and Measurements* (O&M) [32] and the *Sensor Model Language* (SensorML) [33]. In this article we particularly focus on SOS, SensorML and O&M as they are the main specifications involved in the exchange of most sensor data between clients and servers. We consider in our experiments versions 1.0.0 of SOS and O&M and version 1.0.1 of SensorML, because although newer versions of SOS and O&M have been recently approved (as of April 2012), the older ones are still widely used.

SOS-based services provide access to observations from a range of sensor systems, including remote, in-situ, fixed and mobile sensors, in a standard way. The information exchanged between clients and servers, as a general rule, will follow the O&M specification for observations and the SensorML specification for descriptions of sensors or system of sensors (both referred by the term *procedure*). Observations in SOS are grouped into *observation offerings*. An observation offering is a set of observations related by some criteria (e.g., procedure's location). SOS services expose a set of public operations, some of them are mandatory (*core profile*) and others are optional (e.g., *transactional profile*). The core profile is composed by three operations: *GetCapabilities, DescribeSensor*, and *GetObservation*. *GetCapabilities* allows clients to access metadata about the capabilities provided by the server. *DescribeSensor* allows to retrieve descriptions of procedures. *GetObservation* is used to retrieve observational data from the server. This data can be filtered using several parameters, such as procedures, observed phenomena, location, time intervals and instants. The *transactional profile* offers support for data producers to upload observations into SOS servers. Using *RegisterSensor* and *InsertObservation* operations, data producers can register its sensor systems and insert observations into the server, respectively.

The service interfaces and data models in SWE fit nicely in the creation of information systems according to service-oriented architectures (SOA). The main SOA design principles such as loose-coupling between service implementations and interfaces, independence, reusability and composability, encourage the use of SWE specifications and data models in such information systems [14,34]. Therefore, these specifications such as SOS services and O&M data models are becoming common artifacts in the design and creation of SOA-based applications addressing the integration and management of observations and sensor networks. However, in our opinion, their application to the mobile realm is limited because of the large amount of exchanged information, which often exceeds the processing capabilities of mobile phones. The need to reduce data communication is then a crucial aspect, which inevitably relates to data formats used in communication protocols.

XML (eXtensible Markup Language) is likely one of the most widely used formats in data communication on the Web. It has been adopted as the most common form of encoding information exchanged by Web services [35,36]. Kay attributes this success to two reasons [35]. The first one is that the XML specification is accessible to everyone and it is reasonably simple to read and understand. The second one is that several tools for processing XML are readily available. Another reason is that because of its agnosticism the language can be used in almost any domain, and being text-based can be very helpful for debugging purposes. Despite its popularity XML is considered a highly verbose language, which increments unnecessarily the amount of network traffic and storage space occupied by exchanged data represented in this format [20–25]. In this regard, several attempts have been made to overcome this problem such as the use of alternative encoding formats [21–23,25] or the use of compression techniques [37–39]. In this context, *compactness* and *processing efficiency* are competing requirements because the smaller the messages transmitted, the less resources are spent in data transmission, but this may require the use of more processing power if data must be compressed and decompressed. The choice of using compression or not must be carefully considered because it has been proven that wireless communication can be much more expensive than computation in terms of energy consumption [40].

An alternative that is gaining a lot of attention is the *Efficient XML Interchange* (EXI) format [41]. EXI is a W3C's recommendation that is intended to provide “… *a very compact representation for the Extensible Markup Language (XML) Information Set that is intended to simultaneously optimize performance and the utilization of computational resources*”. EXI encodes XML data using a binary format to reduce its verbosity. As stated by [25], a binary format typically has more favourable size and memory properties, hence it is the preferred option for in-memory representation, storage, and transmission. EXI does not offer direct interoperability with XML, but it can be examined, stored, or transmitted as XML format when necessary. It has a *schema-informed* mode that allows users to make use of available schemas to improve compactness and performance. This format also includes the option to apply additional compression through the DEFLATE algorithm (RFC 1951) [42]. EXI has been reported to have very good compression rates and performance by the research literature [37,38,43] and by W3C itself [44].

Another option is JSON (JavaScript Object Notation)(http://www.json.org), which is one of the most popular alternatives to XML because of the easiness to be read and serialized to Javascript variables and to overcome some security limitations present in some browsers [45,46]. Like XML, JSON is a text-based format which can be very useful for debugging purposes. The attention gained by this language as an alternative to XML has been reflected in the research literature [21–23]. The arguments in favour of JSON are that it is simpler than XML, it has less overhead (*namespace information, tags, etc.*), and basic information items of any XML document (element and attribute names, character information items, *etc.*) can be easily mapped to a JSON document.

## 3. Related Work

With the basic concepts and definitions introduced in Section 2, in this section we overview related work from different fields which are relevant for our experiments. First, we present strategies to reduce data communication in ubiquitous sensing, followed by a discussion of previous work dealing with XML performance and the use of alternative formats.

### 3.1. Strategies to Reduce Data Communication in Ubiquitous Sensing

In the context of ubiquitous sensing, transmission of huge volumes of sensor data over the Internet consumes network resources excessively. Efficiency in data communication represents a big issue which has been addressed from different perspectives. The use of caching techniques is a widely adopted strategy to mitigate the amount of exchanged data, as proven by the Web itself. For example, a model to establish the conditions under which caching at the client and proxy side reduces the average response time of a requested document in a Web-based traffic application was presented by [47]. Agent-based systems have been also explored by several authors as a strategy to reduce data communication in ubiquitous sensing [48,49]. These authors proposed similar approaches based on agent-based systems to eliminate needless data communication through implementing data processing capabilities nearby or within sensor nodes. Such agents were installed in a sensor node to handle sensor data, expand data processing capabilities over such sensor nodes, and reduce communication traffic. In the case of [49], agent systems were implemented as web services to be accessed via request-response operations encoded in XML format.

The above works [47–49] put emphasis in three crucial aspects: (i) the need to reduce communication data; (ii) sensor nodes and devices are mainly exposed as web services; and (iii) exchanged data is encoded in XML-related formats. Furthermore, experiments as ours to explore efficiency and performance in data communication by exploring suitable encoding formats become relevant for sensor network systems, services and applications regardless of their application domain.

### 3.2. Performance Analysis for XML Processing and Alternative Formats

Numerous articles have been addressing the topic of XML processing performance. For example, the impact of XML processing in the context of web servers and databases was analysed in [50,51]. These works stated that XML processing is a performance bottleneck in several kinds of applications. An extensive review of XML processing in the context of SOAP-based web services was conducted by [20]. The review analysed different techniques to improve performance in XML serialization (e.g., [52,53] cited by [20]), parsing (e.g., [54–56] cited by [20]), and deserialization (e.g., [57,58] cited by [20]). The use of compression in the context of SOAP was also included in that review, highlighting the study presented in [59] which concluded that traditional compression methods for XML-based documents might not always be appropriate for SOAP messages, because these are of relatively small size (a few kilobytes), in comparison with XML-based documents.

In the last few years, several performance studies have been conducted to evaluate the use of alternatives to XML for data representation in mobile or embedded devices. These studies compared XML with JSON [22,23], and other binary alternatives (e.g., *Fast Infoset* (http://fi.java.net) [21], BXML (*Binary Extensible Markup Language*) [38], EXI [43]). Some of them also included compression tools, such as gzip (http://www.gzip.org) [39] and bzip2 (http://www.bzip.org) [37]. All the studies including XML and JSON coincided that the latter offers an important reduction in message size and better performance [21–23]. Regarding binary alternatives, EXI showed better compression rates than Fast Infoset and bZip2 [37], gzip [37–39], and BXML [38]. Unfortunately, the studies including binary formats and compression tools did not measure processing performance.

W3C performed an extensive performance evaluation of an EXI implementation: AgileDelta's Efficient XML 4.0 (http://www.agiledelta.com/product efx.html) [44]. The experiments evaluated, on the one hand, EXI to *gzipped* XML (XML + gzip) and ASN.1 PER (*Abstract Syntax Notation One, Packed Encoding Rules* [60]) regarding compactness. On the other hand, a second set of experiments compared EXI without compression to XML, and EXI with compression to gzipped XML in terms of encoding and decoding speed. The results showed that EXI files were more compact than gzipped XML regardless of document size, document structure or the availability of schema information. Similarly, EXI produced much more compact data than ASN.1 PER. EXI without compression was 14.5 times faster than XML on average decoding speed. When compression was used EXI was 9.2 times faster than gzipped XML. For average encoding speed EXI was 6 times faster than XML. With compression, it was 5.4 times faster than gzipped XML.

Studies on performance for SWE standards are still few. Particularly for SOS services, in a previous work [61], we conducted a performance analysis that processed sensor data in an Android mobile phone and a desktop PC environment. The results only included XML data processed locally and showed that processing times in mobile devices were around 30 to 90 times slower than those for desktop environments.

This article tries to explore an area that has not been included in the previous works: performance analysis on real mobile phones of large sensor datasets encoded using SWE standards and considering alternative encoding formats. Most of the work exposed above used small XML messages that range from just a few bytes to about 200 KB, except for the W3C's EXI evaluation that was performed on desktop settings. Moreover, just a few of them explored EXI beyond the reduction of size that can be achieved, and only two were targeted to real mobile phones [21,22].

## 4. Data and Methodology

In this section we present the description of the experiments that measure the performance cost of exchanging sensor-related information. Compared with related work in Section 3, we go one step further and build our experiments on a wide range of datasets in terms of size and encoding formats. We explore the use of alternative protocols such as JSON and EXI to experimentally analyse the reduction of size of exchanged data and how the use of these alternative formats affect performance.

### 4.1. Selected Datasets

In our analysis we build a set of datasets with data captured from SOS servers available on the Internet [62]. Data files contain varied information captured by sensors related to meteorological variables such as *temperature, pressure, seawater salinity*, and *rainfall*. These files have been selected to cover a large range of message sizes since we are especially interested in measuring the performance penalty of processing large datasets in mobile phones.

The datasets are described in Table 1 and are grouped according to the type of data they contain (See *description* column). The first three datasets (CAPS, SD and OBS) are consumed by mobile applications, and they illustrate the situation when smartphone applications download data from a server. The last two (RS and IO) reflect the opposite case when sensor-related data is uploaded to a server. These two groups cover the most frequent situations in ubiquitous sensing: data consumption and provision for mobile applications.

For each XML file in each dataset, we create three other files as follows:

- *A JSON file*: Each XML file is converted to JSON using *JSONLib* (http://json-lib.sourceforge.net). Namespace-related information is removed manually from the converted files.
- *An EXI file*: XML files are converted to EXI with default parameters (*bit-packed alignment, no compression, no schema information*) using *EXIficient* (http://exificient.sourceforge.net)
- *A compressed EXI file (EXI-C)*: Using again *EXIficient*, XML files are converted to EXI but keeping its compression capabilities on.

As a result, each dataset is encoded using four different formats, which allows us to measure data size variations and to observe how performance is affected by each format. We do not consider the EXI's *schema-informed* mode because the library used for Android do not support it.

### 4.2. Methods

Apart from defining different datasets and encoding formats, our experiments are extended with two more variables to be as much realistic as possible. We perform our experiments with two different mobile terminals and over two types of communication links (Wi-Fi, 3G). We describe next the metrics and the experimental environment used for the performance study.

#### 4.2.1. Metrics

The main metric to measure performance is *execution speed*, which includes local parsing time on the mobile devices and time spent on data communication with the server. It does not take into account processing tasks at the server side, such as handling requests and generating responses, as we are more interested in the performance from the smartphone perspective. We measure *execution speed* over three scenarios: parsing time from memory (*Local scenario*), parsing time from HTTP source using a Wi-Fi connection (*Wi-Fi scenario*), and parsing time from HTTP source using a 3G connection (*3G scenario*).

In the first scenario, we measure parsing time without considering disk transfer or network delays. To accomplish this, information is first loaded into memory before being processed. These values are compared later to measurements including communication delays (Wi-Fi and 3G scenarios), which allow us to identify the influence of parsing and communication in the overall execution times. According to [63], despite advances in network performance, the time required to access shared resources on a local network remains about a thousand times greater than that required to access resources that are resident in local memory. The time, of course, will be even larger if resources are accessed over the Internet and using wireless technologies.

Calculating execution speed of Java programs in a modern system, such as Android-based smartphones, is a difficult task mainly due to the existence of multiple factors that may alter the final result, such as cache memories, multi-threading, background processes, *Just-In-Time compilation* (JIT), or garbage collection. These factors cause that different executions of the same program may lead to very different results. For this reason, we used the methodology presented by [64], as it provides a statistically rigorous approach to mitigate the effect of all these factors on execution time. The methodology allows to calculate *steady-state performance*, which is the normal action of the application once all its classes are loaded in memory and, in case where available, the JIT compiler has also done its job and the application is supposed to run without major interferences of other factors [64]. It attempts to cope with non-deterministic errors that may affect measurements, by executing iterations of the instrumented code until a consecutive number of measurements (*k*) showing minimal variation is found. Variability is calculated using the *coefficient of variation (COV)* (standard deviation divided by the mean), which is a normalised measure of dispersion of a probability distribution. Mean and confident interval for the mean are calculated for the *k* values. We apply this method to Local and Wi-Fi scenarios with *k* = 10 and *COV* = 0.05. The value of *COV* was determined experimentally as we were unable to obtain measured values with lower variability. A 95% confident interval is calculated for the mean using the Student's *t* distribution. In the 3G scenario we cannot apply this methodology because of limitations of the data plan used by the smartphones (200 MB/month at full speed). In this case, we execute a *warmup* iteration and calculate mean values and confidence intervals for the next 3 iterations.

Additional measures are taken to reduce possible sources of interferences with the instrumented code such as disabling background data synchronization in the smartphones, and stopping manually several applications commonly included in Android or supplied by the phone manufacturer that are continuously executed in the background, such as messaging services and email clients, location services, *etc.*

#### 4.2.2. Experimental Environment

CAPS, SD, and OBS datasets are accessible from an Apache HTTP server (http://httpd.apache.org) that emulates a real SOS server. The HTTP server runs in a 2.26 GHz Dual Nehalem Quad Core CPU, with 24 GB DDR3 RAM w/ECC, and offering download and upload speeds of up to 100 Mpbs. The server is located in a data center in Dallas, Texas. The (emulated) SOS server manages *GetCapabilities, DescribeSensor*, and *GetObservations* requests returning pre-existing response files. For the case of data provision (RS, IO), the server emulates *RegisterSensor* and *InsertObservation* requests by copying the messages' content to a server folder.

For each of the encoding formats we use streaming parsers to support data parsing tasks. We used the XMLPull (http://www.xmlpull.org) parser available in Android for XML, the streaming parser included in GSON (http://code.google.com/p/google-gson/) for JSON, and we ported the *StAX API* (*Streaming API for XML* [65]) provided by *EXIficient* to Android for EXI. The choice of using streaming parsers was made to avoid unfair comparisons between APIs that incur additional tasks, such as creation of application data structures, or performing data type conversions beyond the task of parsing itself. In addition, the use of streaming APIs is preferred for networking applications, as information can be processed as it is received and memory is used much more efficiently [66]. All the parsers considered in our experiments return a very similar stream of events that allow reading the content of each data entity in a similar manner (e.g., XML element, JSON object).

As mobile terminals we use two Android-based smartphones: HTC Desire and Samsung Galaxy SII (SGS2). The HTC Desire smartphone, released in March 2010, has a 1 GHz CPU, 576 MB of RAM, and runs Android OS v2.2. The SGS2 mobile phone was released about a year later with a dual-core 1.2 GHz Cortex-A9 CPU, 1 GB of RAM, and running Android OS v2.3.4. We selected two devices with different processing power to attempt to measure, although very roughly, how much improvement could be expected by using more powerful devices.

In the Wi-Fi scenario, smartphones connect to a 802.11 g access point with a theoretical speed of up to 54 Mbps. During our experiments we monitored signal strength which ranged from −57 to −72 dBm. A connection with these values can be considered as having a good signal strength. Changes in signal strength levels can have great impact on TCP throughput, and low signal levels are more susceptible to random disturbances [67]. We also calculated approximate values for *round-trip time* as the time spanned from the moment a request is sent to the server to the moment the response starts to arrive at the client application. These values were above 150 ms and include, in addition to the actual latency of the underlying network, the software overhead of the implementation of HTTP and network protocols in Android.

In the 3G scenario, an UMTS/HSDPA connection is used, with a download speed advertised by the carrier of up to 7.2 Mbps. Upload speed values are not provided by the carrier. Similarly, signal strength was monitored during the experiments with values ranging between −60 and −65 dBm, which can be also considered as good signal levels. Round-trip times were above 500 ms and showed great variability. These values can be considered as normal, as previous experiments with several network carriers reported median round-trip times between 300 ms and 500 ms at the TCP level [68]. Authors of those experiments also reported that measurements taken at different times and locations vary widely, even for a single carrier, resulting in transfer rates lower than advertised rates.

The experiments in the networked scenarios are executed in a fixed location (Castellón de la Plana, Spain) hence variations in the conditions of the experiments because of devices' mobility do not need to be considered.

## 5. Results

In this section we present the result of the performance experiments. Because of the extension of the results, we present in detail those for CAPS, OBS and IO datasets. The results obtained for the SD dataset were very similar to those of small files in CAPS. Similarly, the results for RS had a lot in common with small files in IO.

### 5.1. CAPS Dataset

Table 2 summarises the information related to size and *content density* of the files in this dataset. *Content density (CD)* is calculated as the percentage of an XML document that is “*actual data*” (attribute and element values), in contraposition to the portion that is “*structure*” (namespace information, tags, *etc.*). According to size and content density, files are classified as follows [69]:

- High CD (Cat. I): content density > 33%, data predominates.
- Low CD: content density < 33%, these are documents with highly structured data and are further separated in:
  – Large (Cat. II): > 100 KB
  – Small (Cat. III): between 1 and 100 KB
  – Tiny (Cat. IV): < 1 KB

**Figure 1.**
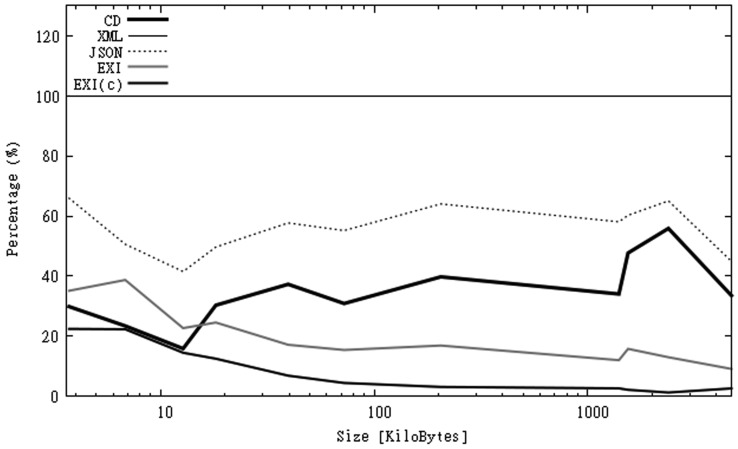
Percentage of size of capabilities files using different formats.

From the numbers in Table 2 we can see that six of the CAPS files have a high CD. The rest of the files belongs to Cat. III. The percentage of reduction achieved by alternative formats along with the content density of each file is represented in Figure 1. In this figure we can observe that by using JSON we can obtain a 40%–60% size reduction. By using EXI, in the best cases, we obtain a 90% reduction without compression and a 98% reduction with compression. The compression ratio increases as files are larger. In the case of JSON, it is obvious that the percentage of reduction cannot fall below the value of content density, as all the “*actual data*” in the XML file must be also included in the JSON file.

#### 5.1.1. Local Scenario

**Figure 2.**
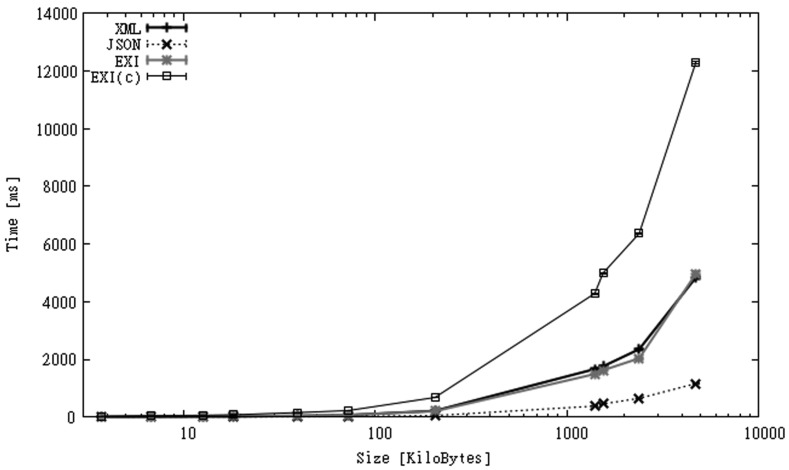
Parsing time for HTC smartphone (CAPS dataset).

**Figure 3.**
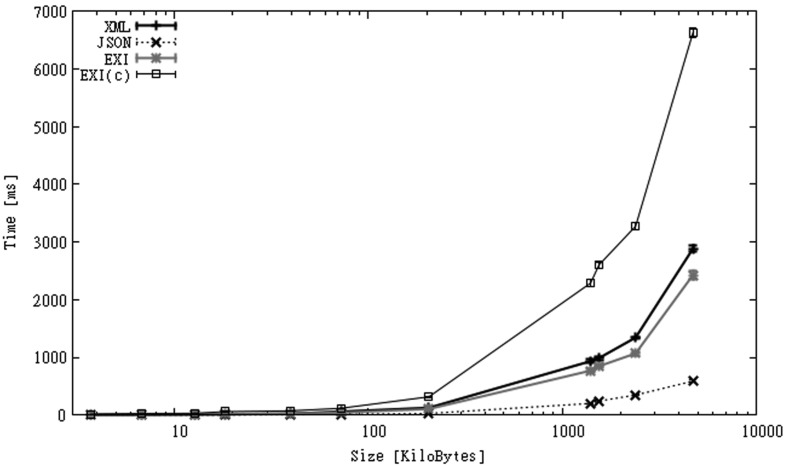
Parsing time for SGS2 smartphone (CAPS dataset).

As mentioned before, local parsing time is the time taken to process a file without considering disk transfer or network delays. Figures 2 and 3 show parsing times for CAPS in both mobile phones. In the figures, *x-axes* represent the size of the original XML files, while *y-axes* represent parsing times in milliseconds. For the HTC phone (Figure 2) we observe that JSON presents the best processing times, with EXI and XML having very similar results, and EXI-C, as expected because of the need to decompress information, being the slowest option. For the SGS2 mobile (Figure 3) the relative difference between the formats is almost the same but the time values are smaller than for the HTC phone. The execution times in the SGS2 phone are 20% to 60% faster than in the HTC phone. In all cases the execution times seem to increase linearly with file size. In this scenario, using JSON is about 3 to 8 times faster than using XML or EXI, and 10 to 20 times faster than using EXI-C.

A study of the impact of network latency on users of interactive applications was presented in [70] (cited by [71]). This study measured users' experience and satisfaction as a function of application response times. Authors categorized subjective impression of response times as follows: *crisp* (<150 ms), *noticeable to annoying* (>150 ms and <1 s), *annoying* (>1 s and <5 s), and *unbearable* (>5 s). According to this classification parsing small CAPS files can be integrated in an interactive mobile application without producing *annoying* or even *noticeable* delays. But for larger files this is only possible if JSON were used. Fortunately, streaming parsers allow information to be shown to users as it is processed, improving the responsiveness of the application. As Android offers a multi-threaded environment, a thread can parse a capabilities file while another one displays the part of the information already processed, e.g., the list of observation offerings.

#### 5.1.2. Wi-Fi Scenario

The Wi-Fi scenario includes the time taken to download sensor-related data over a Wi-Fi connection. In these scenario, round trip times are higher than parsing times for small files (<100 KB) in all formats, except for EXI-C. Hence, time spent on communication represents most of the overall execution times. When larger files are transferred the ratio between communication and parsing time varied greatly between the formats. For example, communication takes between 6 and 15 times more than parsing for JSON files, between 2 and 5 times for XML files, between 0.8 and 1.3 times for EXI files, and between 0.15 and 0.3 times for EXI-C.

**Figure 4.**
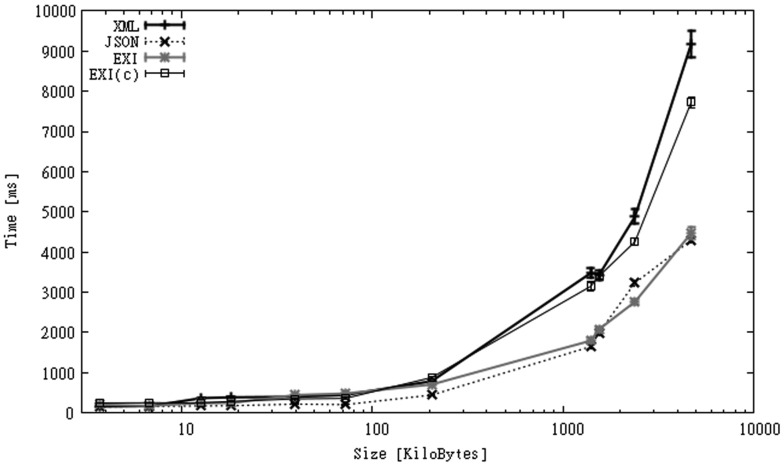
Processing times over a Wi-Fi connection for SGS2 smartphone (CAPS dataset).

Figure 4 shows the overall processing times for the SGS2 smartphone. Numbers for the HTC follow a similar trend but are 40%–80% larger. Under the network conditions of the Wi-Fi scenario, XML is the format with worst execution times, as it spends a lot of time transmitting much larger files. EXI-C shows slightly better results as larger parsing times are compensated by much smaller communication times. JSON and EXI outperform XML and EXI-C, being almost twice faster for files above 100 KB. Even when communication delays are considered, parsing and data transfer do not seem to be an obstacle in terms of execution speed when small files are processed, as for all formats these values are consistently below 1 s. Once more, additional measures have to be taken to not harm application responsiveness when large files are processed.

#### 5.1.3. 3G Scenario

The last scenario uses a 3G connection as communication link. In this case, we exclude the largest file in CAPS because if included, the overall data to be transmitted would exceed the limitations of the data plan used by the 3G connection.

**Figure 5.**
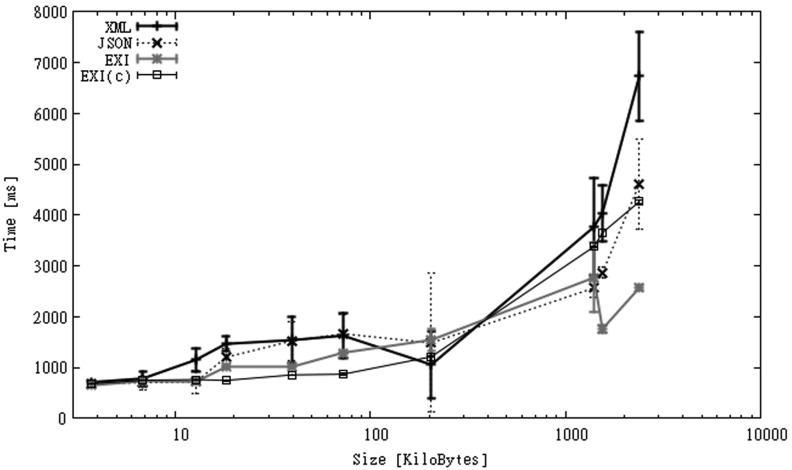
Processing times over a 3G connection for SGS2 smartphone (CAPS dataset).

In this scenario round-trip times, around 500 ms, are even higher than parsing times for EXI-C small files. Because of this and the much smaller size of EXI-C files, the overall times for EXI-C are the best when small files are processed. Although counterintuitive at first, this result makes perfect sense. The burden brought by decompression is tolerable if files are small, because most of the overall times is spent on communication. XML presents again the worst results and on average EXI shows the best. Figure 5 shows the overall processing times for the SGS2 smartphone. The measurements for the HTC smartphone are not shown to simplify exposition. In general, time spent on communication when using 3G was between 1.1 and 7 times slower than in the Wi-Fi scenario.

The lack of control we have over the networking conditions in this scenario, such as signal strength or bandwidth, along with the low number of iterations executed do not allow to generalize the results presented above. Nevertheless, we consider that they reflect what might be expected under less reliable networking conditions: formats with smaller size will be favoured (EXI and EXI-C) even when more CPU time is needed in the device to process them.

### 5.2. OBS Dataset

**Figure 6.**
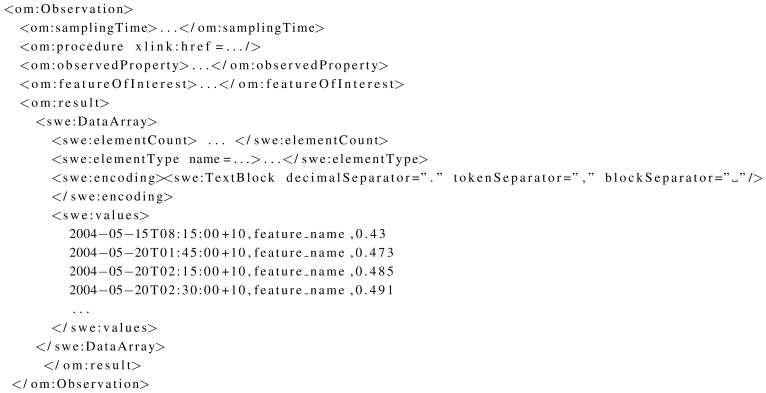
Observations encoded as a block.

The OBS dataset contains files encoded in O&M. Table 3 shows the size of these files for each format, as well as their content density. The values for the content density, almost 100% for larger files, is the most distinctive attribute of these files. This happens because observation values are typically encoded in an *om:Observation* element that packages a group of values that shares some metadata in a single block (Figure 6). This allows to reduce the size of XML messages in comparison to the use of more specialised observation types (e.g., *om:Measurement*) [62]. The block of observations represents almost the total size of larger files. As a consequence, no reduction in size is achieved by using JSON or EXI. On the other hand, EXI-C still presents excellent compression rates (80%–96%).

For this dataset we calculate the parsing times for JSON and EXI, but we decided to exclude them for the Wi-Fi and 3G scenarios. We made this decision because the only advantage of using JSON, having faster parsing times, is measured in the first part of the experiments. On the other hand, EXI does not offer any important advantage as it has shown similar parsing times to XML and the size of EXI files is not much smaller for this dataset. In addition, we only present results for the SGS2 smartphone as performance differences with the HTC phone are similar to what we have shown in the previous section.

#### 5.2.1. Local Scenario

**Figure 7.**
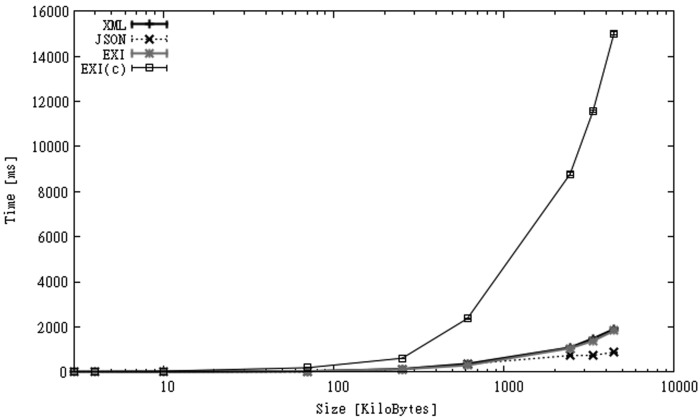
Parsing time for SGS2 smartphone (OBS dataset).

Parsing times for OBS files present a similar behaviour that for CAPS files (Figure 7). Again, JSON is the format that shows lower processing times, being consistently between 1.4 and 5 times faster than XML and EXI. The main difference with OBS files is that time spent on decompressing EXI-C files is considerably larger for this dataset, being up to 25 times slower than JSON. JSON, XML and EXI parsers spend most of the time reading the string value containing observations, and as a consequence they have few extra overhead (parsing tags or object names, processing namespace information, *etc.*). The SGS2 smartphone is 20%–60% faster than the HTC phone for all formats.

Encoding observations as a single string value presents an unwanted side-effect. It is impossible to start processing these values until the whole block of observations has been parsed. This does not allow to process information incrementally, as mentioned in previous sections as a solution to improve application responsiveness. As a consequence, when large files are processed, delays will be annoying for users despite the format used, and they can even be unacceptable in the case of EXI-C.

The option of using more specialised observation types, such as *om:Measurement*, where individual values are separated and are accompanied by its own metadata section, does not seem feasible for handling large volumes of data. In a small experiment, we converted the three largest files in OBS to use *om:Measurement* resulting in a 10 to 20 times increase of the file sizes. Exchanging 30 to 90 MB files over wireless connections does not sound like a good alternative in this case.

#### 5.2.2. Wi-Fi and 3G Scenario

**Figure 8.**
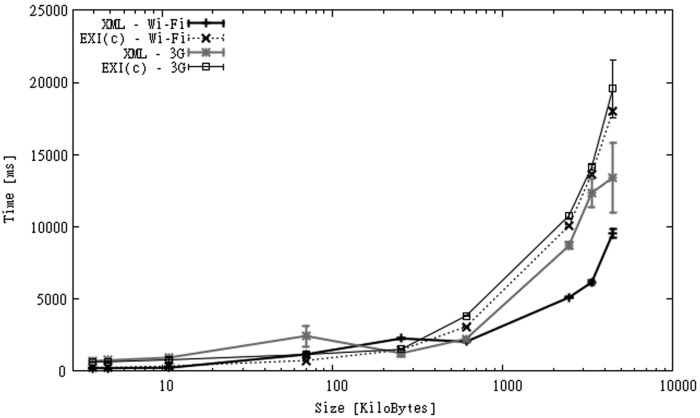
Processing times over Wi-Fi and 3G connections for SGS2 smartphone (OBS dataset).

**Figure 9.**
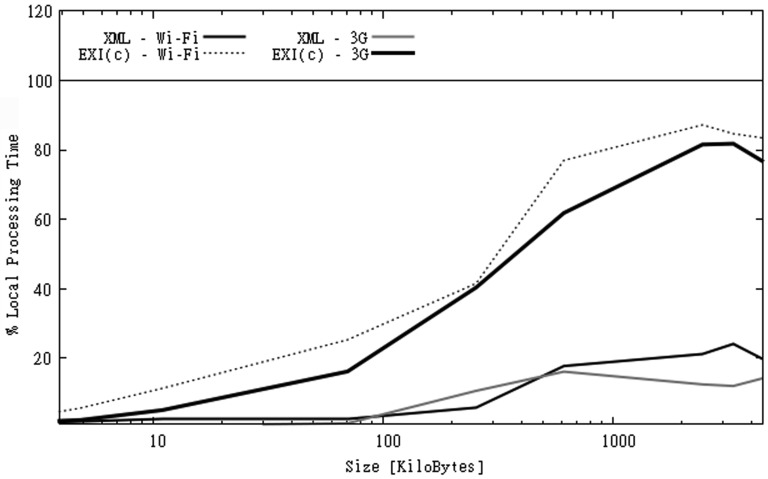
Percentage of time spent on parsing/decompression and communication for SGS2 smarthphone (OBS dataset).

Figure 8 shows the overall execution times for both scenarios. Unlike files in CAPS, the time used to decompress EXI-C data was not compensated by larger communication times of other formats in most of the cases. Communication times for larger EXI-C files were only a fraction of the time spent on decompression (Figure 9). In Figure 8 we can also observe that larger XML files transmitted over 3G shows even better overall execution times than EXI-C files transmitted over Wi-Fi. On the other hand, for small files the use of compression improved the performance for both types of communication links.

### 5.3. IO Dataset

Experiments with the IO dataset differs from those presented before in which data is generated/captured by the phone itself or other sensor(s) that could be attached to or communicating with it, and sent to the server. In this case, we measure the ability of mobile phones to generate the required messages in the given formats. Messages generated in the experiment contain synthetic values of a measured phenomena (e.g., *temperature*). We build messages with a different number of measurements as reflected in Table 4, trying to cover different possible scenarios, such as data transmitted continuously (e.g., in a near real-time application), or data that is stored temporally on the device because of the lack of connectivity or the unavailability of efficient connection links (e.g., it is more efficient to upload larger amounts of data only when Wi-Fi connections are available).

Table 4 also shows the size of files in IO for each format, as well as their content density. Similarly to the case of observation files, the content density is almost 100% for large files and the reduction in size achieved by JSON and EXI is minimal. On the other hand, with EXI-C we achieve compression rates above 98% for files larger than 100 KB.

#### 5.3.1. Local Scenario

Instead of measuring parsing time, in this case we measure *serialization time, i.e.*, the time taken to convert application data in a message in the required format. In this scenario the resulting messages are serialized to the device's main memory. All the experiments for the IO dataset assume that application data is available as string values. The reason for such assumption is that both XML and EXI documents are created using the StAX API, which only allows strings to be specified as attribute or element values. As data type conversion may require a large portion of parsing or serialization times [25], we considered a better option to perform this conversion incrementally when this data is stored locally on the device and not when it is sent to the server. We also build JSON messages differently, rather than using GSON we take advantage that these files are text-based and build messages just by concatenating string values.

**Figure 10.**
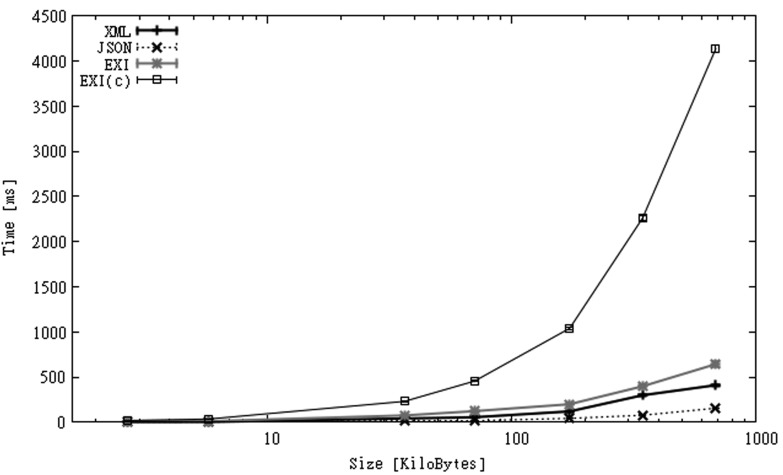
Serialization times for SGS2 smartphone (IO dataset).

Serialization times are shown in Figure 10. These values are very similar to those of OBS, with the difference that time is spent on building the messages and not on parsing them. Once again, JSON is processed faster than the rest of the formats. Although in this case similar executions times could be obtained for XML if we had built messages in the same way, by concatenating string values and not using the XML parser. EXI-C is 15–30 times slower than JSON, 5–10 times slower than XML, and 3–6 times slower than EXI.

#### 5.3.2. Wi-Fi and 3G Scenarios

As we did for the OBS dataset, for these scenarios we only include the results for XML and EXI-C. We have shown previously that because of the way observations are encoded JSON and EXI offer few advantages over XML regarding size reduction and time spent on communication.

**Figure 11.**
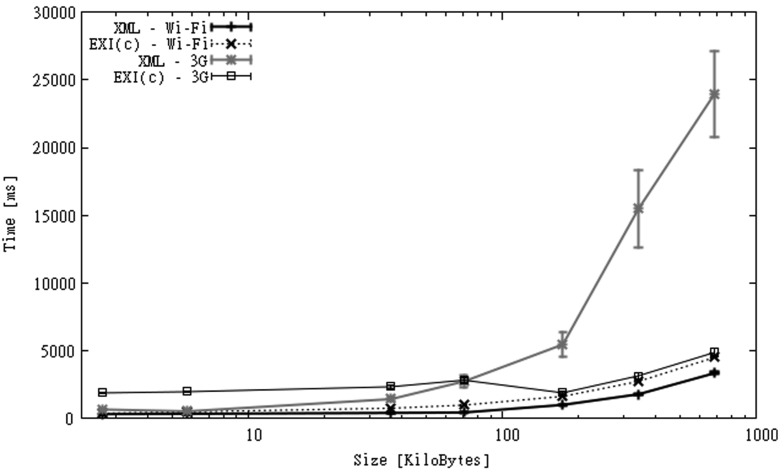
Processing times over Wi-Fi and 3G connections for SGS2 smartphone (IO dataset).

When a communication link is considered we measure the time taken to build and send the message to the server, and to receive an acknowledgment of receipt. The results for Wi-Fi and 3G links are shown in Figure 11. The figure shows that in the case of Wi-Fi, XML performs better than EXI-C, as larger processing times of the latter are not compensated by larger communication times of the former. When a slower connection link is used, the use of compression seems to be a better option. Upload links are expected to be much slower than download links [68], for this reason the potential gain in terms of execution time of sending smaller messages is higher. The figure shows that when uploading messages above 100 KB, the use of EXI-C exhibits much better execution times than XML. This advantage seems to grow quickly as the message size increases.

### 5.4. Summary and Discussion

In the experiments presented in the previous sections we have observed that the use of alternative formats to XML may be used to improve performance of mobile SWE applications. Regarding reduction of data size, JSON allowed reductions between 40% and 60% of the original XML files, while EXI without compression produced a reduction that ranged from around 80% to 90%. The problem with these formats was that they failed to produce a significant reduction for files with very high CD (above 90%) such as OBS and IO files. On the other hand, EXI-C showed excellent results for all files, with reductions ranging between 80% and 98% regardless of the content density of the files.

Regarding parsing times, JSON presented the best results, being 1.4–8 times faster than using XML or EXI, and 10–25 times faster than using EXI-C. Serialization times showed a similar trend. Parsing and serialization times for XML and EXI were very similar for all datasets, with some advantage for XML in serialization. According to previous experiments (see Section 3.2) faster processing times for EXI should be expected, nevertheless that was not the case in our experiments. A possible reason could be that the code of the EXI parser used has been ported to Android without any important modification, thus it is not optimized to be executed in a resource-constrained device. The option of using compression led to an important performance penalty to local processing, being up to 30 times slower than JSON for serialization. Execution times in the SGS2 smartphone were regularly 20%–60% faster than those for the HTC phone.

Processing times for small files (original XML file < 100 KB) of any of the datasets showed that this task can be integrated in an interactive mobile application without producing annoying or even noticeable delays, but for larger files this is only possible if JSON were used. When large files are processed the responsiveness of these applications can be improved by showing the information as it is processed. However, this method cannot be applied to OBS and IO files because they encode observations as blocks of values, preventing them to be processed incrementally.

When communication links were considered, communication times seemed to have a large impact on the overall processing times, taking longer than local computation for some of the formats, especially when no compression was involved. Nevertheless, when the quality of communication links decreased (load, maximum bandwidth, *etc.*), the use of compression started to perform better than XML and even the rest of the formats, which make the option of using compression an alternative that is worth considering, despite the performance penalty imposed on the client-side. Another point in favour of EXI-C when compared with XML is that in the cases where XML performed faster (Figure 8), the gap between them was substantially reduced when most powerful hardware was used, which is a good sign considering how fast phone hardware is evolving. Last, trading more local processing for less transmission time may have a positive impact on battery life because, as stated by [72], energy per instruction executed by phone's CPUs is dropping faster and continue to drop, while the energy used by communication hardware will likely drop at a far slower pace.

## 6. Conclusions

In this article we have presented a performance analysis of using SWE standards as data communication protocols in smartphone applications to consume and produce environmental sensor data. Our experiments were aimed to analyse to what extent the performance problems related to transmitting and processing potentially large messages encoded using XML can be alleviated by using alternative uncompressed and compressed formats such as JSON and EXI.

Our results suggest that using EXI with compression (EXI-C) greatly reduce the size of exchanged messages, but adds a high overhead to processing times in the mobile phones. Nevertheless, it can be an appealing alternative if information is exchanged over very slow or unreliable communication links. This option seems to be also favoured by the increase of processing capabilities of mobile phones and the drop of the amount of energy consumed per instruction executed. Under certain conditions, EXI showed a very good trade-off between size reduction and processing times, even when it does not use compression, which implies less energy consumption. The disadvantage of using this alternative is that it does not reduce the size of observation blocks, which is a major drawback because exchanged information is expected to be composed of observation values in its majority. This problem could be solved by using a different way to encode these values where strings, timestamps and measurement values were not mixed. A pragmatic trade-off could be to use EXI for CAPS, SD, and small observation files, and to use EXI-C only when a large set of observation values must be exchanged or when information is transmitted over very slow or unreliable communication links.

In terms of compactness and data reduction, JSON provides in files with low-medium content density a size reduction of 50% on average. In the case of very high content density files, the reduction rate is far less important as the content density value is a lower bound for this value. Nevertheless, because it presents faster parsing times and it can be seamlessly integrated in Web-based applications using JavaScript, its use in these applications could bring benefits to SWE applications when they do not handle large volumes of observations or network bandwidth is not an issue.

In summary, the encoding format used in data communication in ubiquitous sensing scenarios through smartphone applications is clearly a determining factor, among others, for improving performance. The experiments presented in this article may help application developers to figure out the possibilities of the interplay of encoding formats and performance on data communication to enhance application responsiveness and user's satisfaction in ubiquitous sensing applications.

## Figures and Tables

**Table 1.** Description of selected datasets.

**Dataset**	**Description**	**Role**
Capabilities (CAPS)	These files contain metadata of the server such as name, keywords, information about provider, and list of available observation offerings.	Consumption
Sensor Descriptions (SD)	SD files contain descriptions of procedures as defined by the SensorML specification. The files contain information such as location, measured phenomena, *etc.*	Consumption
Observations (OBS)	These files, in O&M format, contain measurements of a specific phenomenon captured by a procedure.	Consumption
Register Sensor (RS)	RS files contain a sensor description that must be added to an SOS server.	Provision
Insert Observations (IO)	These files contain observation values that must be inserted in an SOS server.	Provision

**Table 2.** Size of files (KBs) for each format and content density of the original XML files (CAPS dataset).

	**XML**	**CD (%)**	**JSON**	**EXI**	**EXI-C**
0	3.73	29.87	2.46	1.31	0.84
1	6.83	23.44	3.46	2.64	1.53
2	12.75	15.89	5.29	2.90	1.85
3	18.15	30.32	9.02	4.46	2.27
4	39.57	37.30	22.83	6.79	2.73
5	72.39	30.90	39.94	11.17	3.23
6	205.27	39.78	131.45	34.70	6.45
7	1,400.36	34.05	813.22	168.65	37.50
8	1,545.22	47.71	930.81	244.26	33.71
9	2,388.27	55.87	1553.77	311.14	30.31
10	4,710.21	33.55	2116.46	430.42	125.26

**Table 3.** Size of files (KBs) for each format and content density of the original XML files (OBS dataset).

	**XML**	**CD (%)**	**JSON**	**EXI**	**EXI-C**
0	3.91	14.32	2.28	1.08	0.78
1	4.84	17.80	1.92	1.25	0.83
2	10.99	68.91	8.54	8.00	2.29
3	70.25	59.46	55.40	44.36	4.34
4	254.63	61.42	192.22	134.91	20.49
5	613.47	99.24	610.40	609.03	20.85
6	2458.48	99.81	2455.41	2454.09	105.76
7	3348.86	99.86	3345.79	3344.44	136.70
8	4459.57	99.81	4453.84	4450.90	281.56

**Table 4.** Size of files (KBs) for each format and content density (CD) of the original XML files (IO dataset).

	**# of Observations**	**XML**	**CD (%)**	**JSON**	**EXI**	**EXI-C**
0	10	2.67	31.77	1.62	1.46	0.75
1	100	5.75	68.29	4.69	4.54	0.77
2	1, 000	36.51	95.01	35.46	35.30	0.91
3	2, 000	70.69	97.42	69.64	69.48	1.03
4	5, 000	173.23	98.95	172.17	172.02	1.35
5	10, 000	344.13	99.47	343.07	342.92	1.86
6	20, 000	685.92	99.73	684.87	684.71	2.85
